# Subcellular Architecture of the *xyl* Gene Expression Flow of the TOL Catabolic Plasmid of Pseudomonas putida mt-2

**DOI:** 10.1128/mBio.03685-20

**Published:** 2021-02-23

**Authors:** Juhyun Kim, Angel Goñi-Moreno, Víctor de Lorenzo

**Affiliations:** a Systems Biology Department, Centro Nacional de Biotecnología-CSIC, Campus de Cantoblanco, Madrid, Spain; b Centro de Biotecnología y Genómica de Plantas (CBGP, UPM-INIA), Universidad Politécnica de Madrid (UPM), Instituto Nacional de Investigación y Tecnología Agraria y Alimentaria (INIA), Campus de Montegancedo-UPM, Pozuelo de Alarcón, Madrid, Spain; c School of Computing, Newcastle University, Newcastle Upon Tyne, United Kingdom; University of Washington

**Keywords:** *Pseudomonas putida*, *xylUW*, *xylX*, mRNA localization, specific DNA locus, TOL plasmid, biodegradation, *m*-xylene, mRNA, nucleoid, stress adaptation, transcriptional regulation, translational control

## Abstract

Despite intensive research on the biochemical and regulatory features of the archetypal catabolic TOL system borne by pWW0 of Pseudomonas putida strain mt-2, the physical arrangement and tridimensional logic of the *xyl* gene expression flow remains unknown. In this work, the spatial distribution of specific *xyl* mRNAs with respect to the host nucleoid, the TOL plasmid, and the ribosomal pool has been investigated. *In situ* hybridization of target transcripts with fluorescent oligonucleotide probes revealed that *xyl* mRNAs cluster in discrete foci, adjacent but clearly separated from the TOL plasmid and the cell nucleoid. Also, they colocalize with ribosome-rich domains of the intracellular milieu. This arrangement was maintained even when the *xyl* genes were artificially relocated to different chromosomal locations. The same held true when genes were expressed through a heterologous T7 polymerase-based system, which likewise led to mRNA foci outside the DNA. In contrast, rifampin treatment, known to ease crowding, blurred the confinement of *xyl* transcripts. This suggested that *xyl* mRNAs exit from their initiation sites to move to ribosome-rich points for translation—rather than being translated coupled to transcription. Moreover, the results suggest the distinct subcellular motion of *xyl* mRNAs results from both innate properties of the sequences and the physical forces that keep the ribosomal pool away from the nucleoid in P. putida. This scenario is discussed within the background of current knowledge on the three-dimensional organization of the gene expression flow in other bacteria and the environmental lifestyle of this soil microorganism.

## INTRODUCTION

**FIG 1:**
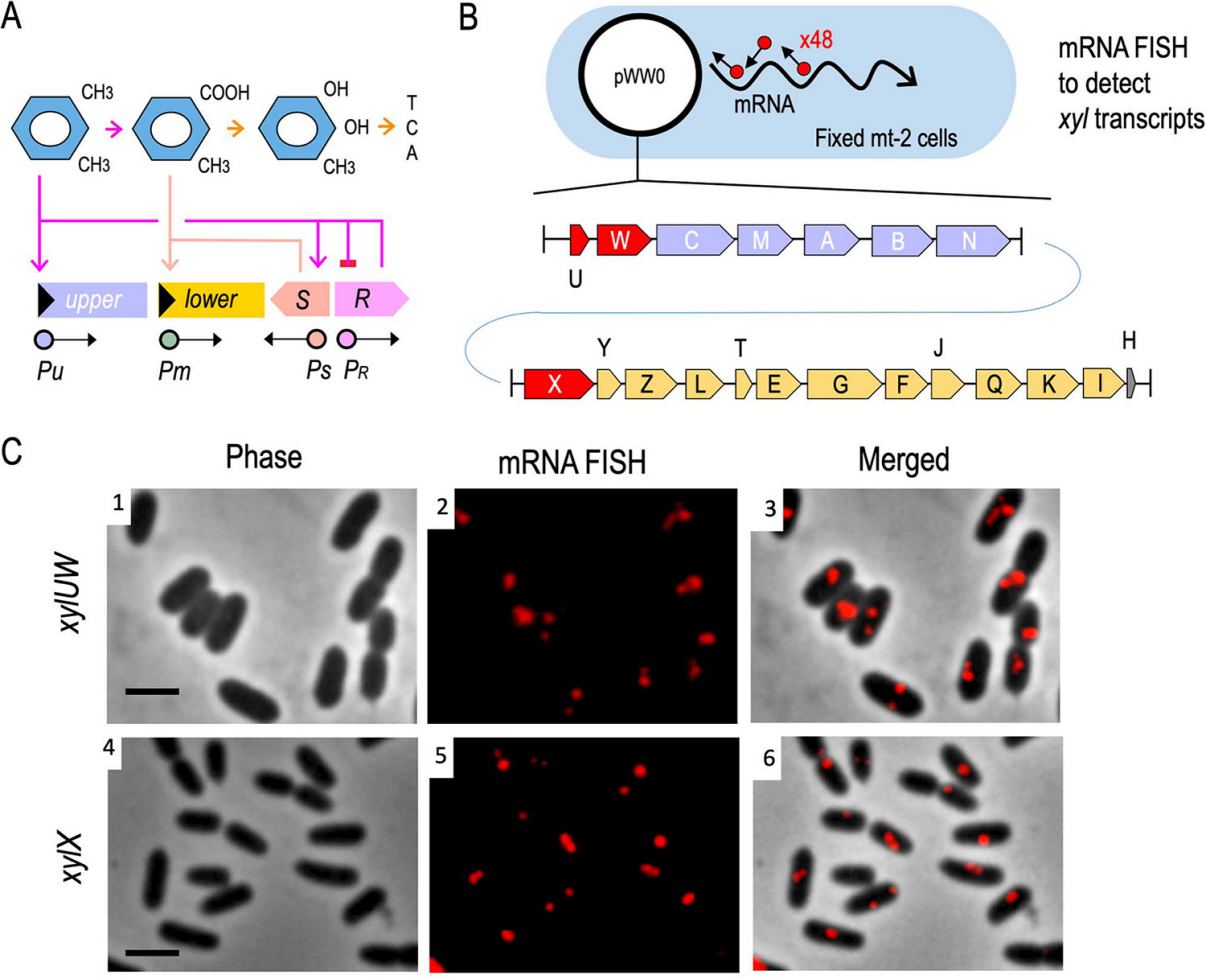
Interplay between metabolic and regulatory components of the TOL network and visualization of transcripts from either the upper or the lower operons of the system. (A) In the presence of *m*-xylene, XylR (expressed from *P_R_*) activates both the *Pu* promoter (which transcribes the upper pathway operon) and the *Ps* promoter, stimulating expression of the *xylS* gene. Also, XylR expression is negatively auto-regulated. In turn, the XylS protein induces the lower operon by activating the *Pm* promoter with its effector 3-methylbenzoate (3MBz), an intermediate metabolite of *m*-xylene biodegradation. (B) Scheme of the fluorescent *in situ* hybridization (FISH) approach adopted to visualize mRNAs of the TOL operons of plasmid pWW0. For this experiment, P. putida mt-2 strain was grown to mid-exponential phase in succinate-supplemented minimal medium and then exposed for 2 h to saturating vapors of *m*-xylene to activate transcription of the *xyl* genes. After fixation of samples with formaldehyde, the FISH experiment was performed with either the upper (*xylUW*) or the lower pathway (*xylX*) probe set, which included a mix of 48 synthetic oligonucleotides tagged with a red fluorophore and complementary to each mRNA sequence. (C) FISH on *xylUW* and *xylX* transcripts, as indicated. Phase contrast (panels 1 and 4); mRNA-red signals (panels 2 and 5); and composite images (panels 3 and 6) are shown. Scale bar, 2.5 μm.

The TOL system, encoded by plasmid pWW0 of Pseudomonas putida mt-2, is to this date the most thoroughly studied example of a biodegradative system in soil microorganisms. The primary function of this catabolic device is enabling carrier bacteria to grow on toluene, *m-*xylene, *p-*xylene, and other related aromatics through a set of enzymes encoded by upper and lower plasmid-borne operons (Fig. 1A; 1–3). While catabolic traits of this sort are not uncommon in many other environmental isolates, what makes the TOL system special is the extraordinary regulatory intricacy that controls expression of the *xyl* genes and their high-level interplay with the host’s physiological regulons. The many mechanisms unveiled over the years in this respect seem to include nearly every device known in the prokaryotic world for controlling the flow of gene expression (4–6). This state of affairs has made the TOL plasmid and its bearer (P. putida KT2440) a beneficiary of the suite of conceptual and material tools of contemporary systems and synthetic biology (7, 8). In particular, the wealth of experimental data on expression of the *xyl* genes has enabled the understanding of the cognate regulatory network as a complex device that processes inputs into outputs following a layer of logic gates implemented with promoters, transcriptional factors, and small RNAs (sRNAs; 9, 10). It could thus be argued that we know at this point much about the genetically encoded “software” of the system and the relational logic that rules its performance. In contrast, we know virtually nothing of the physical arrangement of the “hardware” that sustains the same process. In particular, the gross spatial disposition of the molecular actors that execute the transfer of information from the *xyl* genes to production of catabolic enzymes is unknown.

The same types of questions have been tackled in other prokaryotic systems. There seem to be at least two different patterns of transcript localization. In one scenario, the mRNA remains close to the site of transcription. This is the case of the mRNAs of *groESL* and *creS* of Caulobacter crescentus and *lacZ* of Escherichia coli, which appear to remain in the vicinity of the corresponding genomic DNA loci from where they are transcribed (11). This suggests that for mRNAs to be translated they need to be associated with ribosomes while being produced (as the prevailing view of generalized transcription-translation coupling would imply) or shortly after their creation by RNA polymerase (RNAP; 11, 12). An alternative scenario involves migration of mRNAs to specific spots of the cytoplasm for translation close to the site(s) where the gene products are needed. While this setting argues against generalized transcription/coupling translation, there is solid evidence of its occurrence, e.g., the *bglGFB* operon, whose products were located in subcellular localizations where cognate proteins were expected to function (13). This example may not be anecdotal, as transcriptome-wide scale studies of mRNA localization in E. coli revealed that a large share of transcripts were found in cell domains (e.g., membranes, cytoplasm, poles) that coincided with the functions of the corresponding proteome (14, 15). Furthermore, the mRNA of archetypal membrane proteins LacY and TetA are distinctively close to the cell envelope (16, 17). In other instances, a signal-recognition particle (SRP) is involved in the movement of mRNAs to the membrane, as translation of some mRNAs encoding inner membrane proteins produces a signal peptide that recruits such SRP and the complex leads the transcript to its intracellular address (15, 16, 18). Finally, Rho-dependent transcription termination in Bacillus subtilis is somewhat weak, and “runaway,” i.e., untranslated, mRNAs are abundant (19). In sum, the fate of each transcript seems to be both gene (i.e., sequence)-dependent and species-dependent.

In this work, we have inspected the localization of mRNAs initiated in the catabolic promoters of the TOL plasmid pWW0 of P. putida mt-2. The starting point for tackling the issue is the earlier observation that RNA polymerase (RNAP) of P. putida fully colocalizes with the chromosomal DNA of the nucleoid while being entirely apart from the bulk of the ribosomal pool (20). This observation suggested that, rather than being coupled to translation *ab initio*, many (if not most) of the mRNAs initiated at chromosomal promoters need to move to ribosome-rich, nucleoid-free domains of the intracellular space for translation. Note that in the case of the *xyl* promoters, transcription initiates in an extrachromosomal element, and therefore the three-dimensional (3-D) itinerary of the corresponding mRNAs could be different. As shown below, by merging genetic analyses with *in situ* RNA-FISH (fluorescence *in situ* hybridization) and DNA-FISH technology, we could faithfully locate the relative positions of the nucleoid DNA, the pWW0 plasmid, the *xyl* transcripts, and the ribosomes to predict the motion of cognate mRNAs through the cell interior. The results exposed an unexpected degree of physical partitioning among the material actuators of the gene expression flow that may help P. putida to deal with its typical environmental settings.

## RESULTS AND DISCUSSION

### mRNAs of catabolic genes expressed from the TOL plasmid are spatially organized.

To visualize specific mRNAs of P. putida mt-2 stemming from the TOL operons of plasmid pWW0, we adopted an RNA-FISH approach (Fig. 1B). To this end, the strain was cultured in M9 minimal medium with succinate as the sole carbon source until the cells reached exponential phase. At this point, the cultures were exposed (or not) to saturating vapors of *m*-xylene to induce transcription of the catabolic *xyl* genes. After 2 h, samples were collected and fixed with formaldehyde for hybridization with specific probes as described in the Materials and Methods. For this, two sets of 48 CAL Fluor Red 610-tagged fluorescent oligonucleotides (20 nt long; Table S2 in the supplemental material) were synthesized that covered, respectively, the leading 1,418 bp of the upper TOL operon transcript, spanning the whole of *xylU* and part of *xylW* (encoding benzyl alcohol dehydrogenase), and the front segment of the TOL lower operon, encompassing 1,203 bp of the *xylX* gene (alpha subunit of toluate 1,2-dioxygenase). After hybridization with these oligonucleotide sets, the samples were washed and red signals inspected with fluorescence microscopy.

As shown in Fig. 1C, distinct, discrete fluorescent foci were clearly noticed under the microscope (1 to 2 per cell) from the cultures subject to *m*-xylene exposure upon *in situ* hybridization with upper or lower pathway-specific oligonucleotides. In contrast, no signals were detected in bacteria grown in M9-succinate without aromatic effector (Fig. S1A). These observations confirmed that the designed probe sets and the methodology were working as expected, as there were no signals that could be attributed to hybridization with DNA. Therefore, the foci appeared to represent upper or lower *xyl* transcripts. To further verify the experimental approach, cells were treated with either toluene (an alternative TOL substrate) or *o-*xylene (a gratuitous inducer of the upper pathway and downstream activator of the lower pathway (21, 22; Fig. S1B). Under these conditions, fluorescent foci for both the *xylUW* and the *xylX* transcripts were observed as expected. In contrast, cells treated with benzoate or 3-methyl benzoate (substrates of the lower pathway) produced signals for *xylX* mRNA only (Fig. S1B). Appearance of the fluorescent signals matched the known regulatory network that rules the interplay between the substrates, regulators, and catabolic operons of the TOL system (Fig. 1A). We could thus safely consider that the foci inside cells shown in Fig. 1C were the result of the bona fide hybridization of the fluorescent probes to specific mRNA of *xylUW* or *xylX*. Closer inspection of the images revealed that no red output ever appeared dispersed throughout the cell, but always as discrete foci (Fig. 1B). Yet, their position in respect to the cell shape varied among cells, with signals located near the center, the poles, the contour, or the septum of the cells (Fig. 1C). We thus set out to characterize this asymmetrical localization of the *xyl* transcripts with respect to the nucleoid occupation and to the TOL plasmid, as explained below.

10.1128/mBio.03685-20.4 FIG S1 RNA-FISH experiments with *xyl* probes applied to P. putida mt-2 cells grown with/without TOL aromatic effectors. Download FIG S1, PDF file, 1.1 MB. Copyright © 2021 Kim et al. 2021 Kim et al. https://creativecommons.org/licenses/by/4.0/ This content is distributed under the terms of the Creative Commons Attribution 4.0 International license.

### *xyl* mRNAs occupy subcellular nucleoid-free regions.

**FIG 2:**
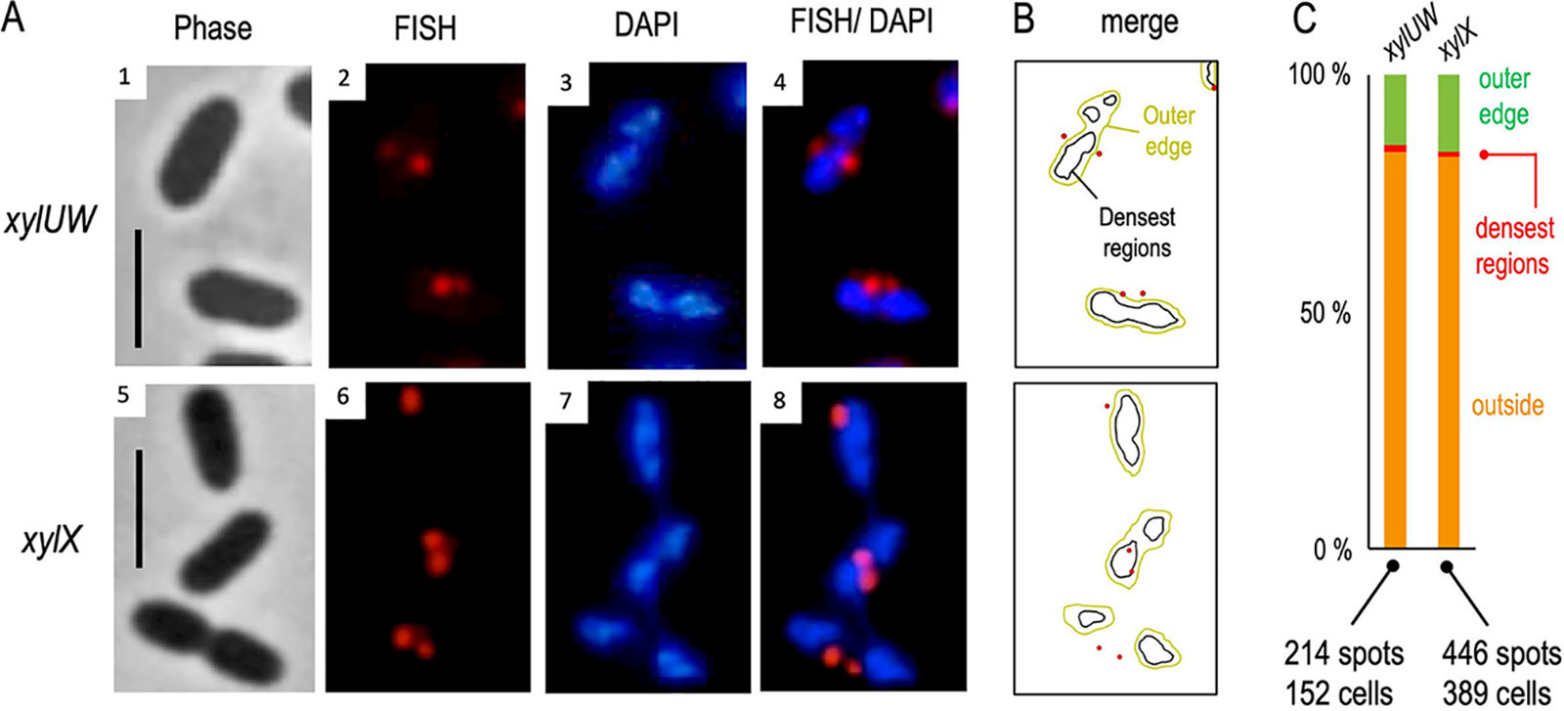
Spatial distribution of TOL catabolic transcripts in respect to the nucleoid of P. putida cells. (A) mRNA signals of *xylUW* and *xylX* were obtained with FISH (panels 2 and 6), while the nucleoid was stained with DAPI (panels 3 and 7). The mRNA-red and the DNA-blue channels were merged (panels 4 and 8) to identify relative positions of the *xyl* transcripts with respect to one another. Phase-contrast images (panels 1 and 5) of the same cells are also shown. Scale bar, 2.5 μm. (B) Application of the image analysis tool CellShape on exemplary cells enabled identification of the contours of each fluorescent signal. The densest regions of the DAPI-stained DNA are delimited with black lines, while relatively less concentrated DNA sections are bounded by yellow lines. (C) Abundance of *xyl* mRNAs with respect to the nucleoid. *xylUW* (214 spots from 152 cells) or *xylX* signals (446 spots from 389 cells) were quantified according to their coincidence with the DNA signal.

In order to identify the relative localization of the *xyl* transcripts with respect to the nucleoid following hybridization with the fluorescent probes, as described above, the bacterial genome was stained with 4′, 6-diamidino-2-phenylindole (DAPI) in cells exposed to *m-*xylene. The results of this procedure with the upper and lower pathway TOL probes (*xylUW* and *xylX*), respectively, are shown in Fig. 2. Images were processed and signals analyzed with the CellShape software. For interpreting these results, note that (i) previous work showed that the DNA of the nucleoid overlaps spatially with the P. putida RNAP (20) and (ii) DAPI binds both chromosomal and plasmid DNA. Inspection of the cells under the microscope (Fig. 2A) revealed that signals stemming from *xylUW* or *xylX* transcripts (red foci) were located in sites inside cells with a low DAPI signal, i.e., in the nucleoid-free regions. In order to quantity the phenomenon, >100 pictures of individual DAPI-stained and fluorescent oligonucleotide-hybridized cells were separately recorded with the blue and red channels of the fluorescence microscope and automatically inspected with the CellShape image analysis tool ((23); Fig. 2B). The outcome of this analysis is shown in Fig. 2C. Very few red foci (<1%) overlapped with the densest DAPI signals, and as little as 15% of fluorescent spots from either *xylUW* or *xylX* RNA were detected at the peripheral regions of genomic DNA. Instead, the vast majority of the remaining foci were situated away from the blue signal (Fig. 2C). It thus appeared that the bulk of TOL transcripts were located within subcellular regions with no or little overlap the DNA signal and therefore virtually devoid of RNAP (20). This suggests that, once formed, *xyl* mRNAs could migrate for translation to a site different from the place where transcription is initiated. Note that the pathway substrate (*m-*xylene) dissolves in the cell membrane (24). Moreover, the *xylM* product (hydroxylase component of the leading pathway enzyme xylene monooxygenase) is located in the membrane (25). It is not uncommon that genomic sites encoding envelope-associated proteins are trans-cribed coupled and cotranslationally inserted to the membrane through a so-called “transertion” mechanism (16). In the case of the TOL transcripts, it appeared instead that movement away from the DNA begins after transcription has terminated and the mRNA has disengaged from the nucleoid. To clarify this, we examined the relative position of the *xyl* mRNA with respect to their actual origin inside the cells (i.e., the TOL plasmid), as described below.

### *xyl* transcripts map near but do not overlay the TOL plasmid.

**FIG 3:**
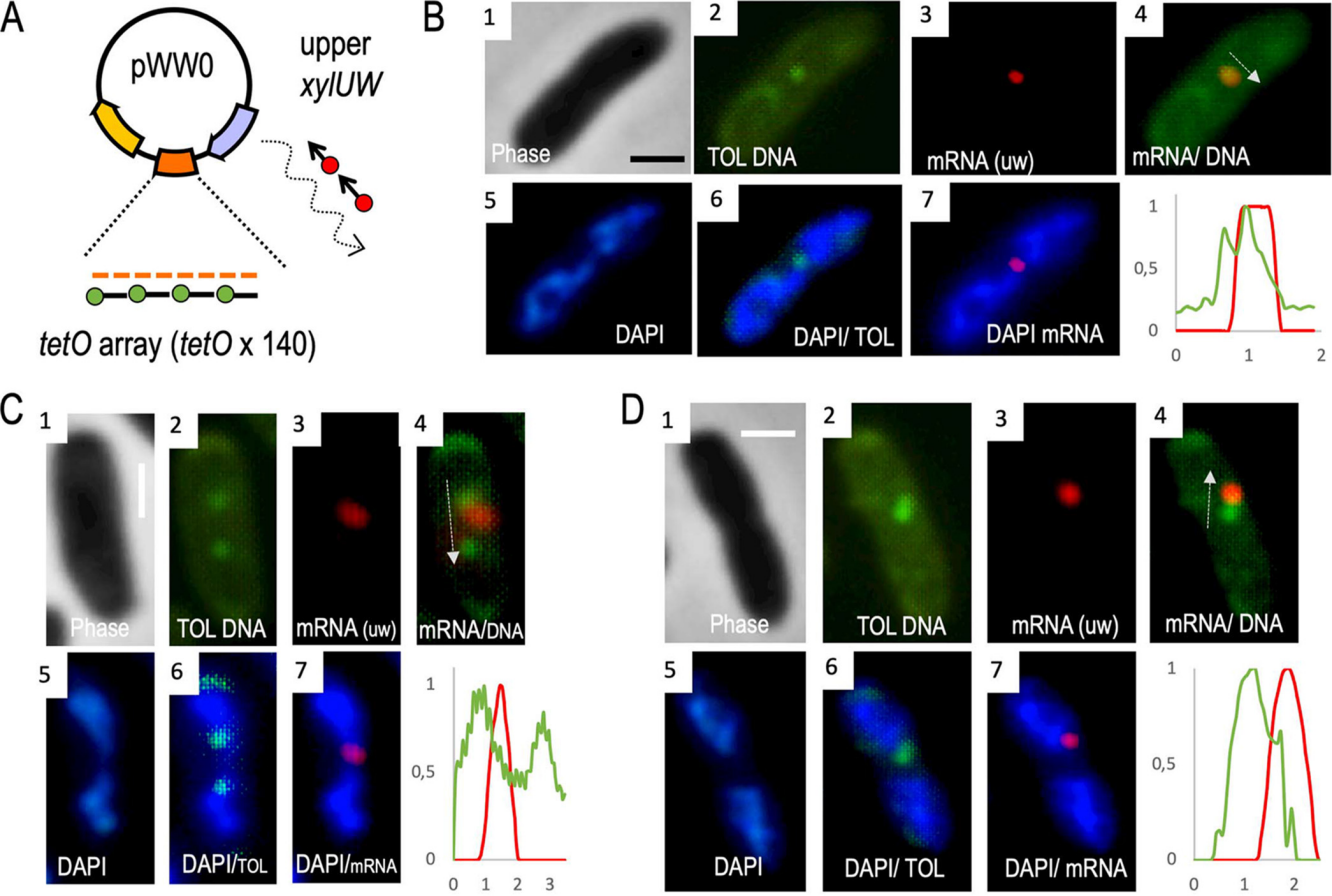
Covisualization of the *xylUW* transcript and the pWW0 plasmid. (A) Sequentially combined RNA-FISH and DNA-FISH was carried out to visualize both the *xylUW* mRNA and the pWW0 plasmid in P. putida cells. Bacteria carried a variant of the plasmid inserted with an array of *tetO* operators in a permissive locus between the upper and lower operons. A green fluorophore-tagged *tetO* probe was used for hybridization in the DNA-FISH procedure. (B to D) P. putida mt-2 (pTOL-*tetO*) strain was exposed to *m*-xylene for 2 h and processed for FISH. A few representative cells are shown, exposing dynamic localization patterns of the mRNA relative to the DNA. Pictures show phase contrast of cells (panels 1), plasmid DNA (green, panels 2), *xylUW* mRNA (red, panels 3), and the nucleoid (blue, panels 5), respectively. Composite images include mRNA/plasmid DNA (panels 4), plasmid DNA/nucleoid (panels 6), and mRNA/nucleoid (panels 7). Relative intensities of mRNA-red and plasmid-green signals were measured and represented in plots in which the *y* axis refers to relative fluorescence intensity and the *x* axis indicates pixel distance projected on the arrow drawn in merged panels 4. Scale bar, 1 μm. As an aside, the images in panels labeled 2 also show that the TOL plasmid exists as a low-copy replicon (∼1 molecule per cell of P. putida).

**FIG 4:**
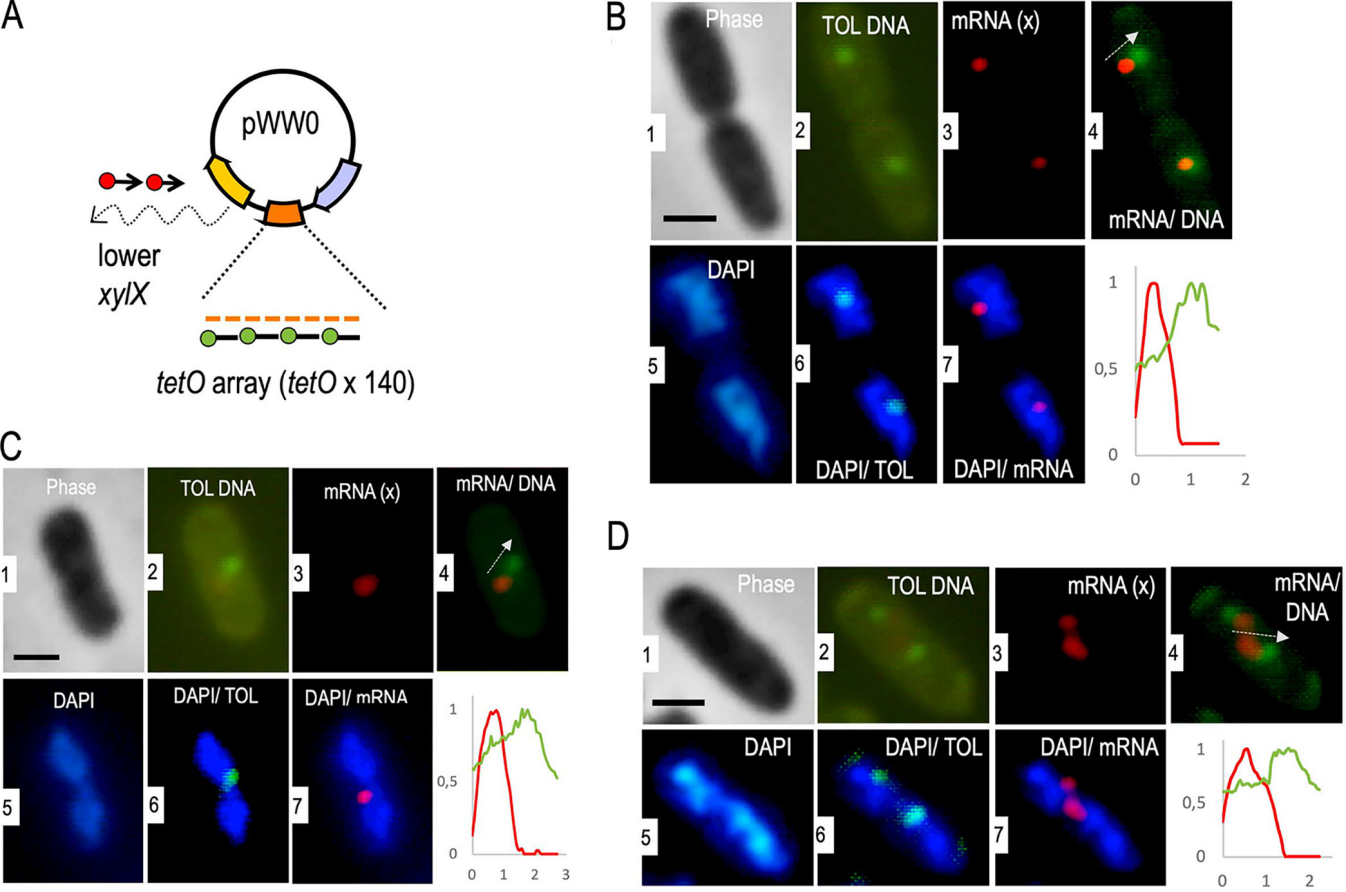
Dual labeling of the *xylX* mRNA and the pWW0 plasmid. (A) The same approach was used as described in Fig. 3A, but red fluorophore-tagged *xylX* probes were used in this case for the combined RNA-FISH and DNA-FISH to visualize both the lower pathway transcripts and the TOL plasmid. (B to D) Cellular location of plasmid DNA versus *xylX* mRNA. Panel tags are the same as those of Fig. 3.

Identifying the subcellular localization of the TOL plasmid within the whole of the DAPI-stained DNA of the P. putida nucleoid required first tagging pWW0 with a different fluorescent label. A 9,679-bp DNA sequence, including an array of tandemly repeated *tetO* operators for the TetR repressor (26), was inserted into a site in the plasmid between and close to the upper and lower operons, i.e., by ORF105 of the current annotation (27; Fig. S2A). The corresponding sequences could then be exposed through FISH with a special type of 6-carboxyfluorescein (6-FAM)-labeled 18-nt oligonucleotides. These encoded the *tetO* operator and bore a distinctive chemical configuration (so called locked nucleic acid [LNA] structure) that increases specificity for target DNA (11; Fig. S2A). Samples were thus first hybridized with *xyl*-specific (red fluorescence) and pWW0-specific (green fluorescence) probes and then stained with DAPI. As a result of this procedure, both red, green, and blue fluorescent signals could be mapped in cells exposed to *m*-xylene, corresponding to *xylUW* or *xylX* mRNA, plasmid, and nucleoid, respectively (Fig. 3B to D and Fig. 4B to D). In contrast, cells grown without the aromatic compound lacked the red signal altogether (Fig. S3A).

10.1128/mBio.03685-20.5 FIG S2 DNA-FISH to visualize the pWW0 plasmid tagged with an array of *tetO* operators. Download FIG S2, PDF file, 0.6 MB. Copyright © 2021 Kim et al. 2021 Kim et al. https://creativecommons.org/licenses/by/4.0/ This content is distributed under the terms of the Creative Commons Attribution 4.0 International license.

10.1128/mBio.03685-20.6 FIG S3 Dual labeling of pWW0 plasmid and *xyl* mRNAs. Download FIG S3, PDF file, 0.9 MB. Copyright © 2021 Kim et al. 2021 Kim et al. https://creativecommons.org/licenses/by/4.0/ This content is distributed under the terms of the Creative Commons Attribution 4.0 International license.

The control images (panels 1 and 2 of Fig. 3B to D and Fig. 4B to D; Fig. S3) show individual bacteria hosting discrete green spots in cells bearing the TOL plasmid variant tagged with the *tetO* array. In contrast, no defined green foci were observed in cells with the intact TOL plasmid devoid of the same array (Fig. S2B). This verified that the *tetO* probe hybridized specifically to the tagged plasmid without significantly interacting with any other DNA of the cells. Further inspection of the pictures indicated that the green signals appeared only once or twice in every cell (in the last case in a symmetrical fashion; Fig. S3). Such a subcellular distribution of the plasmid has been observed before (28) and plausibly reflects the plasmid partition system (29–31). Once the position of the TOL plasmid was determined, by filtering the images with the adequate color channels we could pinpoint the relative localization of pWW0, the nucleoid, and the TOL transcripts.

Fig. 3B to D and Fig. 4B to D show a few examples of cells processed and filtered to locate the *xylUW* (upper pathway) and the *xylX* mRNAs (lower pathway), respectively. Each of these images was not only visually inspected but the signals were also quantified with the CellShape software (23; Fig. S4). In general, the transcripts from either the upper or the lower TOL pathway behaved similarly. The first piece of information resulting from a detailed image analysis (panels 2 and 6 of Fig. 3B to D and Fig. 4B to D) is that the TOL plasmid could be found both in DAPI-intense regions and in the peripheral space of the cytoplasm with little or insignificant DAPI signal. This agrees with the pWW0 predicted segregation system; the presence of plasmid-encoded *parA* and *parB* indicates pWW0 bears a type I partition mechanism (27) through which the plasmid can either detach or remain connected to the chromosome through a ParA-ATP-nucleoid complex (30).

10.1128/mBio.03685-20.7 FIG S4 Single-cell mapping of the *xyl* gene expression flow in P. putida mt-2 (pTOL-t*etO*) cells. Download FIG S4, PDF file, 0.6 MB. Copyright © 2021 Kim et al. 2021 Kim et al. https://creativecommons.org/licenses/by/4.0/ This content is distributed under the terms of the Creative Commons Attribution 4.0 International license.

Inspection of colored spots merged within the contours of single cells exposed two predominant spatial arrangements of the different signals. In one case, red and green spots virtually colocalized (e.g., Fig. 3B), while in most others, the two signals were close to each other but clearly separated (Fig. 3C and D and Fig. 4B to D). It is likely that these two scenarios reflect different stages of transcription of *xyl* genes. In an early stage, mRNA is necessarily tethered to its DNA template in the plasmid and therefore the gene and its transcript occupy the same spot. However, at a later stage, the *xyl* mRNA could move away from the plasmid toward nucleoid-free ribosome-rich domains of the inside of the cell (20). Note that the sizes of the upper and lower TOL operons are ∼8 Kb and 11 Kb, respectively, and, if linearly stretched, the 5′ ends of their full-length mRNAs could be found well away from the plasmid while still tethered to the template. However, it is known that the mRNA from the lower pathway is quite short-lived and starts being degraded before it is fully synthesized (32). Note also that mRNA tends to form secondary structures that shorten the distances between the 5′ and 3′ ends (33). On this basis, we argue that separation of red and green spots in the images shown in Fig. 3, Fig. 4, and Fig. S4 reflects an authentic migration of the TOL transcripts away from their DNA template. The next obvious question was whether this was the result of TOL genes being encoded in a type of plasmid that largely stays in the periphery of the nucleoid (see above). Alternatively, the unexpected localization of *xyl* mRNAs could stem from intrinsic properties of the transcripts themselves. In order to distinguish between these possibilities, we introduced various types of perturbations into the system.

### *xyl* transcripts move away from the nucleoid regardless of their replicon.

**FIG 5:**
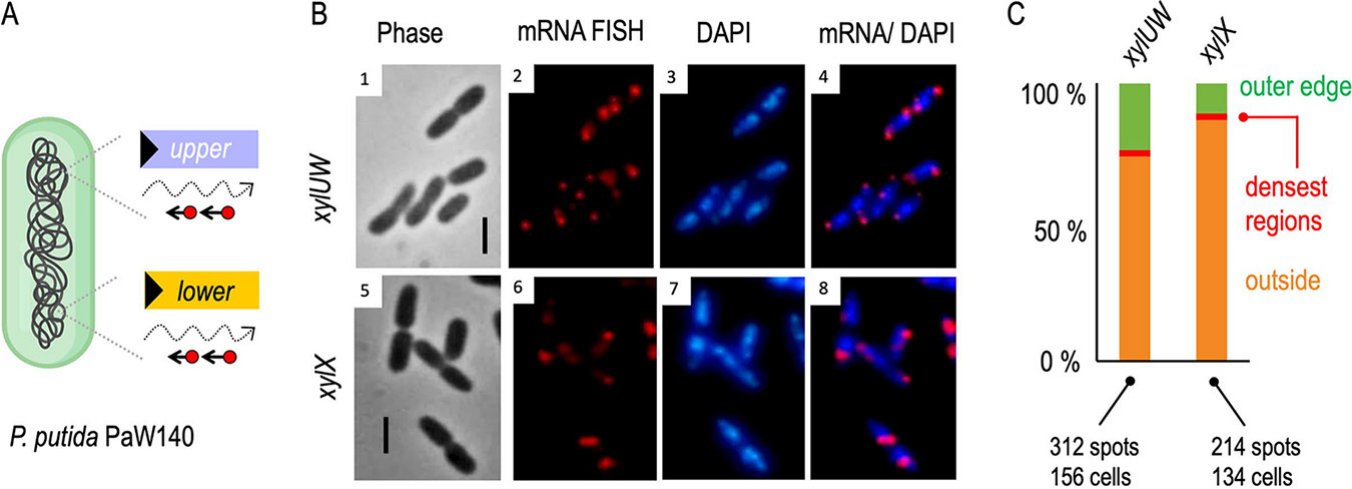
Effect of replicon type on localization of *xyl* mRNAs. (A) The P. putida PaW140 strain, carrying the two operons of the TOL system inserted into the chromosome, was used to identify the distribution of *xyl* mRNAs with the same methods used with the P. putida mt-2 strain above. (B) RNA-FISH-enabled visualization of *xylUW* (panel 2) and *xylX* mRNA signals (panel 6). Cognate phase-contrast images (panels 1 and 5), the nucleoid (blue, panels 3 and 6), and merged pictures (panels 4 and 8) are shown. Scale bar, 2.5 μm. (C) Quantification of mRNA-red foci with respect to the nucleoid occupation. Note that the number of red spots per cell increased by ∼30% in the P. putida PaW140 strain compared to P. putida mt-2 (Fig. 2C).

Although the native physical location of the TOL genes is on plasmid pWW0, the same growth phenotype on aromatic compounds can be brought about when the upper and the lower pathways are placed into the chromosome. This may happen either naturally (34) or by engineering the corresponding DNA segments into the genome of a heterologous host (35). We took advantage of this for exploring whether the position of the *xyl* DNA template affects the spatial distribution of the transcribed RNAs. To this end, we used P. putida strain PaW140 (36), which carries both the upper and lower operons and their cognate regulators in its chromosome (Fig. 5A). The strain was grown and induced with *m*-xylene under identical conditions as before and cells fixed and hybridized with RNA probes to expose *xylUW* and *xylX* transcripts with respect to the bacterial nucleoid. The results in Fig. 5B show the number of foci per cell to increase by ∼30% in P. putida PaW140 compared to the lower numbers in the pWW0-bearing P. putida mt-2 strain (Fig. 2C), quite likely due to a higher transcriptional activity. This is not surprising, as the pedigree of strain P. putida PaW140 involved random chromosomal insertion of TOL genes and selection for the best growth on *m-*xylene, which plausibly favored insertions into regions of high transcriptional activity (37–39). Yet, when RNA-red signals were compared to those of the DAPI-stained nucleoid, most of them were observed away from the denser DNA regions. Specifically, <3% of the red spots of either *xyl* mRNAs overlapped the more compacted chromosome, while 74% of *xylUW* and 87% of *xylX* signals were enriched in the peripheral space of the cell (Fig. 5B and C). Preferential localization of *xyl* mRNAs was thus maintained regardless of the replicon (plasmid versus chromosome) that bears the cognate genes. Moreover, the higher incidence of TOL mRNAs in the nucleoid-free subcellular regions corroborates that *xyl* transcripts move away from their transcription site.

### Exclusion of *xyl* mRNAs from the nucleoid is independent of the transcriptional machinery.

**FIG 6:**
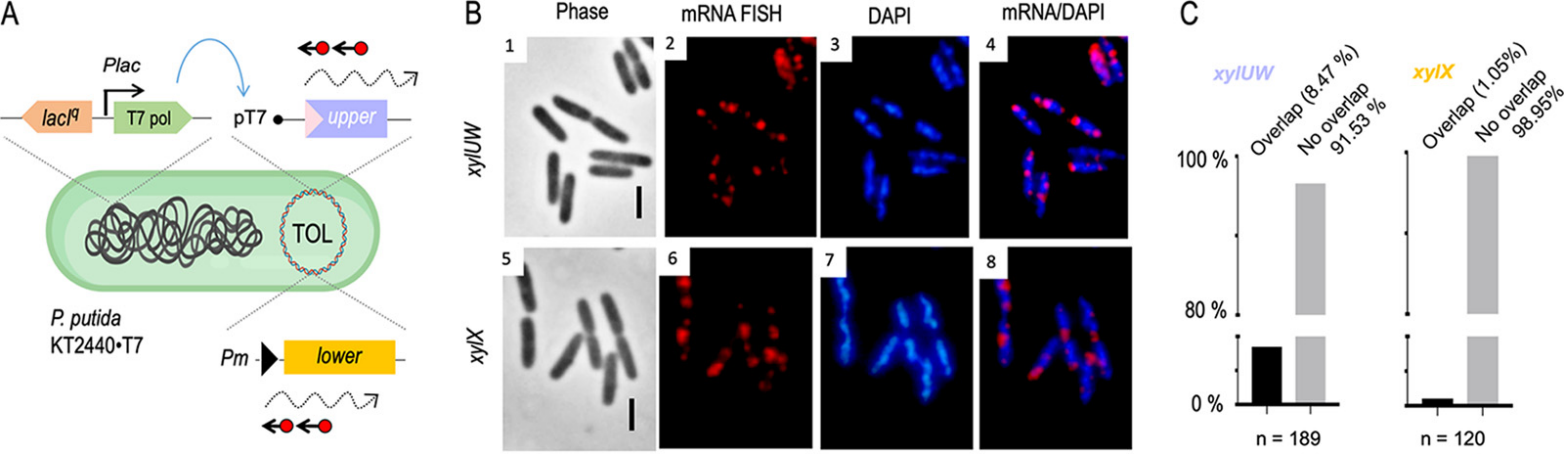
Localization of *xyl* mRNAs transcribed with an orthogonal RNAP. (A) Schematic of RNA-FISH experiments with the modified plasmid pTOL-PuxT7 in P. putida strain KT2440·T7, which expresses T7 RNAP from a chromosomally inserted *lacI^q^/Plac-T7pol* cassette. After 2 h of exposure of cells to *m*-xylene, *xyl* transcripts were visualized with RNA-FISH technology as before. (B) *xylUW* mRNA (panel 2); *xylX* mRNA (panel 6); DAPI-stained DNA (panels 3 and 7); merged pictures (panels 4 and 8); and phase contrast (panels 1 and 5). Scale bar, 2.5 μm. (C) Quantification of mRNA-red and DAPI-blue signal overlap with subpixel precision (>100 exemplary cells were considered for the colocalization analysis).

A second type of perturbation in the architecture of the *xyl* gene expression flow involved complete replacement of the native σ^54^-dependent *Pu* promoter of the upper operon of the pWW0 plasmid (40, 41; Fig. 1A) by a heterologous expression T7 system. The replacement was engineered into the pWW0 plasmid as explained in the Materials and Methods section and the resulting construct (pTOL-PuxT7) was then passed to strain P. putida KT2440·T7 (42), which expresses the T7 RNA polymerase through a genomic insertion of a *lacI^q^/P_lac_-T7pol* cassette (Fig. 6A). As shown in Fig. S5A, the modified plasmid did not support *m*-xylene metabolism in cells without T7 RNAP because it did not transcribe the upper pathway. Due to the leakiness of the *P_lac_-T7pol* born in the P. putida chromosome, some red signals indicative of x*ylUW* expression were detected in samples without isopropyl-β-D-thiogalactopyranoside (IPTG; Fig. S5C). Note also that there were RNA dots stemming from the lower pathway in the wild-type P. putida mt-2 host bearing the modified plasmid pTOL-PuxT7 (Fig. S5B). This is because *m*-xylene-activated XylR caused overexpression of XylS, which in turn suffices to activate the *Pm* promoter even in the absence of any effector (5, 43, 44). As a consequence, when pTOL-PuxT7 was placed in P. putida KT2440·T7, two things happened: (i) *P_T7_* activation elicited formation of both *xylUW* transcripts (Fig. 6, panels 1 to 4) and *xylX* transcripts (Fig. 6, panels 5 to 8) in the complete absence of the aromatic inducers used before; and (ii) cells could grow on minimal medium with *m*-xylene as sole carbon source (Fig. S5A) due to the performance of both the upper and the lower TOL pathways. This made sense within the current model of regulation of the *xyl* operons discussed above (Fig. 1A). But in contrast to the effect of the native effectors in the naturally occurring system, the surrogate control of the upper route by T7 polymerase originated a higher number of distinct foci per cell. While this reflected the strength of the *P_T7_* promoter compared to *Pu* (which propagated into a more potent activity of the *Pm* promoter as well), it is noteworthy that the red signals always appeared focused (i.e., constrained within a subcellular domain) rather that diffused throughout the cytoplasm. Further, they again materialized mostly in the nucleoid-free regions. Automated quantification of signals detected with the red and blue channels in individual cells with pixel precision revealed that 91% of the *xylUW* and 98% of the *xylX* transcripts were excluded from the DAPI-stained field (Fig. 6C). Given that such transcripts originated in single promoters per bacterium and that nucleoid-free domains of P. putida cells are filled with ribosomes (20), it is possible that the images of Fig. 6 reflect the detachment of the transcripts from their promoters (whether *P_T7_* for *xylUW* or *Pm* for *xylX*) and their relocation elsewhere in the cytoplasm for translation.

10.1128/mBio.03685-20.8 FIG S5 Expression of *xylUW* mRNA with the orthogonal T7 system and visualization of the upper transcripts. Download FIG S5, PDF file, 1.2 MB. Copyright © 2021 Kim et al. 2021 Kim et al. https://creativecommons.org/licenses/by/4.0/ This content is distributed under the terms of the Creative Commons Attribution 4.0 International license.

In addition, one important piece of evidence embodied in these experiments is that formation of mRNA foci and apparent motion toward ribosome-rich subcellular sectors occurs regardless of whether the cognate RNAP can (host polymerase) or cannot (T7pol) couple transcription to translation. This rules out that the distinct foci of *xylUW* and *xylX* mRNAs shown throughout this work reflect the action of transcription/translation complexes (the so-called expressome [45, 46]) on the nucleoid surface. Instead, it seems that the untranslated TOL transcripts move away from their promoters, plausibly toward other subcellular locations for translation. But, if that were the case, what physical forces prevent their diffusion throughout the cytoplasm?

### Disruption of intracellular crowding enables *xyl* transcripts to diffuse throughout the cytoplasm.

The data above support that *xyl* mRNAs leave the proximity of the nucleoid toward the ribosome-enriched subcellular domains. Although a role for specific RNA-binding proteins cannot be ruled out as drivers of the process, a simpler explanation is that the corresponding sequences endow the *xyl* transcripts with physical properties that cause them to be quickly discharged from the vicinity of the nucleoid, especially if the transcribed sequences are large. In fact, it seems that smaller RNAs appear uniformly distributed throughout the bacterial cell, while longer molecules typically display more limited dispersion (47). Large mRNAs can hardly colocalize with densely packed DNA regions due to straight physical forces, e.g., excluded volume effects (48, 49). As a consequence, the more crowded the bacterial cytoplasm, the less diffusion there is of cellular components and molecules, logically, in a size-dependent fashion (50, 51).

In order to test whether such a mutual exclusion between different intracellular domains accounted for the unusual behavior of the *xyl* mRNAs, we treated P. putida KT2440·T7 (pTOL-PuxT7) cells with rifampin. Since T7 RNAP is not sensitive to this antibiotic, the upper TOL transcript (*xylUW*) can still be produced, while the *xylX* gene of the lower pathway should not. However, it is known that the diffusion rate of ribosomal proteins is faster (11, 12) and the chromosomal DNA of the nucleoid expands after treatment with this drug (52, 53). Finally, the dearth of ribosomes available for interacting with RNAs due to inhibition of 16s rRNA synthesis (54) may also ease diffusion of otherwise tethered transcripts. As a consequence, rifampin treatment elicits a major change in the partition of the different components of the gene expression flow and causes a less compact intracellular milieu.

**FIG 7:**
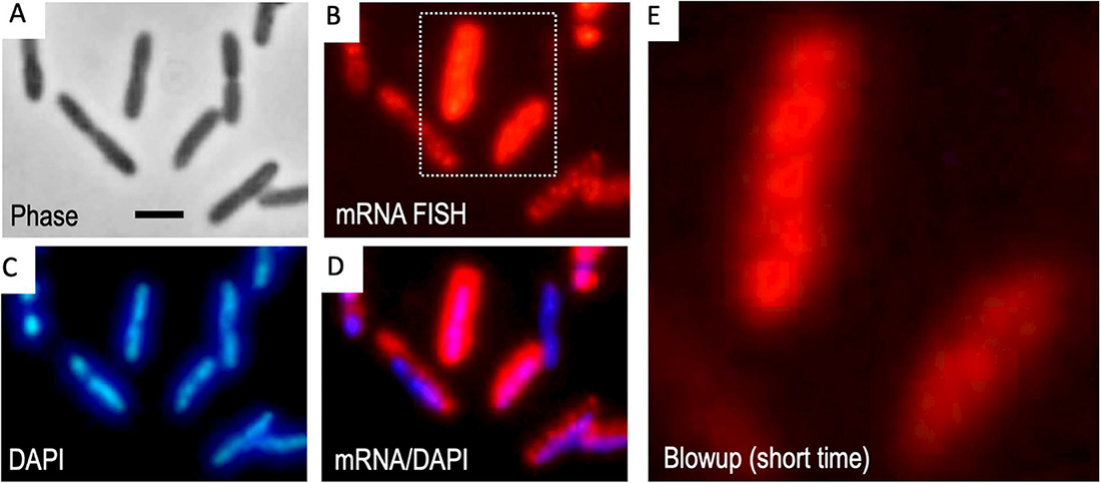
Effect of perturbing macromolecular crowding—by inhibiting bacterial transcription—on the spatial distribution of *xylUW* mRNA. P. putida KT2440·T7 (pTOL-PuxT7) cells were exposed for 2 h to *m*-xylene and rifampin (200 μg ml^−1^) and the intracellular location of *xylUW* mRNA was assessed with RNA-FISH as before. (A) Phase-contrast. (B) Red signals indicate fluorescently labeled oligonucleotides that hybridize to *xylUW* mRNA (note dispersion throughout the cell). (C) DAPI-staining. (D) Merged picture of mRNA-red and DAPI-blue channels. (E) Same image as panel B with a shorter exposure time. Scale bar, 2.5 μm.

After treatment of growing P. putida KT2440·T7 (pTOL-PuxT7) culture for 2 h with the drug and *m-*xylene vapors, the mRNAs were visualized with FISH as before. As expected, we could hardly detect any *xylX* mRNA signal (Fig. S6). In contrast, when cells were hybridized with the *xylUW* probe under the same conditions, red signals did appear (Fig. 7 and Fig. S7). Yet, inspection of individual bacteria revealed a considerable variability in the intensity of the signals and their intracellular distribution. Some cells had their whole contour nonuniformly filled with red color, while others showed a number of discrete red signals with lower intensity (Fig. 7 and Fig. S7). This outcome is not altogether unexpected, as the strong T7 promoter in the plasmid (55) drains cellular resources (56, 57) for the sake of transcription of the upper pathway, thereby causing noise. This effect can be exacerbated in a plasmid, as the loss of gyrase production with the drug and the ensuing accumulation of local supercoiling can lead to stochastic transcriptional bursts (58–60). In any case, we argue that the lack of distinct localization patterns in rifampin-treated cells reflects the diffusion of the TOL transcripts under these conditions. Moreover, some images showed enrichment of *xylUW* mRNAs in the cells’ internal periphery, where the chromosome was otherwise highly compacted (Fig. S7, marked). In these cases, it looked like mRNA was unable to penetrate the denser DNA regions but could freely diffuse into the rest of the cytoplasmic space. Taken together, analysis of images shown in Fig. 7 and Fig. S7 indicated that the *xylUW* transcript of rifampin-treated cells lost its restraint in discrete foci and could then freely circulate, as the cellular crowding had decreased upon antibiotic addition. In sum, the data suggest that under native conditions, *xyl* mRNAs become localized away from the nucleoid because of their entrapment with the translational machinery and the physical forces that determine phase separation between the different components of the gene expression flow. As shown above, if such a separation is perturbed upon rifampin addition, *xyl* transcripts can then circulate through the whole cytoplasmic interior.

10.1128/mBio.03685-20.9 FIG S6 Visualization of *xylX* mRNA in P. putida KT2440·T7 (pTOL-PuxT7) cells after inhibition of bacterial RNAP with rifampicin. Download FIG S6, PDF file, 0.6 MB. Copyright © 2021 Kim et al. 2021 Kim et al. https://creativecommons.org/licenses/by/4.0/ This content is distributed under the terms of the Creative Commons Attribution 4.0 International license.

10.1128/mBio.03685-20.10 FIG S7 Visualization of *xylUW* mRNA in rifampicin-treated P. putida KT2440·T7 (TOL-PuxT7) cells. Download FIG S7, PDF file, 0.4 MB. Copyright © 2021 Kim et al. 2021 Kim et al. https://creativecommons.org/licenses/by/4.0/ This content is distributed under the terms of the Creative Commons Attribution 4.0 International license.

In conclusion, this study shows that *xyl* mRNAs are constrained within subcellular regions with limited mobility rather than being freely diffusible inside P. putida mt-2 cells. Most *xyl* transcripts were detected in the space peripheral to the nucleoid, i.e., in a subcellular domain with virtually no DNA but enriched in ribosomes (20). That TOL mRNAs were detected away from the genetic loci where they originated suggests that they can migrate to translational sites from the 3-D location in the cell where they were synthesized. Our data also show that physical forces and translation are crucial for bringing about this scenario, as it disappeared when in-house transcription was halted to reduce cellular crowding and ribosome engagement. This picture departs from the standard view of transcription-translation coupling, where RNAP and ribosomes get together in the so-called expressome complex (45, 46) which, obviously, must operate in close proximity to the nucleoid’s DNA.

While transcript entrapment is a known mechanism of RNA control in eukaryotes (61–63), the phenomenon as such has not been documented in prokaryotes. However, should transcript diffusion away from the promoter and capture by ribosomes in a different cell location occur, confinement in given spots for translation could become visible. Some mRNAs can indeed travel throughout the interior of E. coli cells (64), and translation inhibition makes RNA remain closer to the nucleoid (47). This suggests that, at least in some cases, ribosomes in fact pull mRNA from the nucleoid toward the rest of the cytoplasm. The reality is that the interplay between the various components of the gene expression flow varies dramatically among bacterial types. In particular, the nucleocytoplasmic (NC) ratio changes greatly from one species to another, originating in cytoplasms with different biophysical properties (65) that affect ribosome mobility and localization. For instance, the high NC ratio of Caulobacter crescentus forces ribosomes toward mRNAs and their translating ribosomes to remain close to their genetic loci in the chromosomal DNA (11, 65, 66). In contrast, the low NC ratio of E. coli allows segregation of ribosomes and mRNA away from their transcriptional sites. *Pseudomonas* spp. also have a low NC ratio (65) and the ribosomes are spatially segregated from the nucleoid (20). It is thus conceivable that TOL transcripts are not coupled to translation as they are produced, but instead move after complete transcription toward ribosome-rich cytoplasmic spots.

What could be the advantage of this setting of the *xyl* gene expression flow in P. putida compared to an alternative subcellular architecture? This species has bona fide *nusA* and *nusG* homologues encoded in its genome. This indicates that transcription/translation coupling can indeed occur (45, 46), although its global incidence compared to E. coli or *Bacillus* (19) is unknown. It may well happen that having movable *xyl* mRNAs eases assembly of the biodegradative complex encoded by the TOL operons for degrading aromatic compounds. This is because the cognate catabolic enzymes, in particular those which are membrane bound (see above), could be synthesized at an optimal location site close to the site of action. Moreover, newly synthesized enzymes could be produced close to each other and enable metabolic channeling (67). Finally, while Rho and transcription/translation coupling helps avoiding RNAP collisions and R-loops—with the downside of polarity—some bacterial types could have solved the traffic jam problem by evolving a different type of termination (19). In fact, interruption of nontranslated transcripts may be detrimental for emergence of new catabolic operons; nonsense mutations in lead genes could prevent expression of the whole cluster and curb evolution of downstream ORFs. These are all of course hypothetical scenarios that deserve further studies.

## MATERIALS AND METHODS

### Culture conditions.

Unless otherwise indicated, E. coli and P. putida were routinely grown at 37°C and 30°C, respectively, in Luria-Bertani (LB) or M9 minimal medium (6 g liter^−1^ Na_2_HPO_4_, 3 g liter^−1^ KH_2_PO_4_, 1.4 g liter^−1^ [NH_4_]_2_SO_4_, 0.5 g liter^−1^ NaCl, 0.2 g liter^−1^ MgSO_4_·7H_2_O) with 10 mM succinate. Bacteria were cultured in 100-ml Erlenmeyer flasks with shaking at 170 rpm with 20 ml of the medium specified for the corresponding experiment. Whenever necessary, kanamycin (Km, 50 μg ml^−1^), ampicillin (Ap, 150 μg ml^−1^), gentamicin (Gm, 10 μg ml^−1^), or chloramphenicol (Cm, 30 μg ml^−1^) was added to cultures of bacterial cells for ensuring plasmid retention and maintenance of manipulated genotype. For the induction of TOL catabolic genes during RNA-FISH experiments, P. putida strains carrying the *xyl* genes either in the pWW0 plasmid or in the chromosome were cultured overnight in succinate-amended M9 and then the bacterial cultures were 100-fold diluted in the same medium and grown until exponential phase (optical density at 600 nm [OD_600_] = 0.3 to 0.5). Samples were next either cultivated further without additional substrate or exposed to vapors of *m*-xylene (1:2 dilution in dibutylphthalate, which is a noneffector for TOL genes) in a flask for 2 h. Additional tests were made by exposing the P. putida mt-2 strain to either vapors of volatile effectors (e.g., toluene or *o*-xylene) or soluble substrates such as benzoate (5 mM) and 3-methylbenzoate (3MBz; 5 mM), as indicated. Transcription of the *xyl* genes by endogenous RNA polymerase was halted by adding 200 μg ml^−1^ rifampin (Rif) to the growth medium of the cognate-sensitive strains.

### Genetic constructs.

The bacterial strains, plasmids, and primers used in this study are described in Tables S1 and S2 in the supplemental material. General methods for DNA manipulation followed standard protocols (68). Plasmid DNA was isolated with commercial Wizard *Plus* SV minipreps DNA purification kit (Promega) or the QIAprep Spin miniprep kit (Qiagen). To replace the *Pu* of the upper operon of the pWW0 plasmid by the T7 phage promoter, we used the seamless allelic replacement method described in reference 69. The delivery plasmid for executing the promoter replacement (pTOL-*Pu*x*T7*) was built as follows. First, the upstream (TS1*^Pu^*, ∼0.5 kb) and downstream (TS2 *^Pu^*, ∼0.5 kb) regions of the pWW0 plasmid around the *Pu* promoter were amplified with primer pairs PuxT7-TS1F/R and PuXT7-TS2F/R, respectively (Table S2). Splice overlap extension PCR (SOEing PCR) was then used as explained in reference 69 to join the amplicon TS1^Pu^ and TS2^Pu^ (including 3′- and 5′- of the T7 promoter sequence with overhang complementarity) by using primer pairs PuXT7-TS1F and PuXT7-TS2R (Table S2). The resulting PCR product was cloned as EcoRI-BamHI segments in the pEMG vector, forming plasmid pEMG-PuxT7, which was kept in E. coli strain DH5α λ*pir*. This plasmid was next delivered to the genome of P. putida mt-2 by triparental mating using the E. coli strain HB101 (pRK600) as helper strain (70, 71), and a cointegrate generated upon selection for Km^R^. The pSW plasmid that expresses I-SceI endonuclease under the *Pm* promoter (72) was then introduced by electroporation into the pEMG-PuxT7-cointegrated strain and thus had resistance for both Km and Ap. The clones were grown in LB medium with Ap (500 μg ml^−1^) and 3MBz (15 mM) to activate the *Pm* promoter, allowing I-SceI expression. The cells were then plated on LB agar and promoter replacement in the TOL plasmid was verified by testing the loss of the pEMG-PuxT7-encoded Km resistance gene. Sensitive clones were further confirmed with PCR with diagnostic T7F/PuXT7-TS2R primer pairs (Table S2). Finally, the resulting plasmid pTOL-*Pu*x*T7* was either maintained in the mt-2 strain or transferred into the KT2440·T7 strain by conjugation, as indicated.

10.1128/mBio.03685-20.1 TABLE S1 Description of bacterial strains and plasmids used in this study. Download Table S1, PDF file, 0.09 MB. Copyright © 2021 Kim et al. 2021 Kim et al. https://creativecommons.org/licenses/by/4.0/ This content is distributed under the terms of the Creative Commons Attribution 4.0 International license.

10.1128/mBio.03685-20.2 TABLE S2 Oligonucleotide primers used in this study. Download Table S2, PDF file, 0.04 MB. Copyright © 2021 Kim et al. 2021 Kim et al. https://creativecommons.org/licenses/by/4.0/ This content is distributed under the terms of the Creative Commons Attribution 4.0 International license.

To label pWW0 with an array of *tet* operators, we inserted the cognate DNA sequence into a dispensable locus of the plasmid. To this end, a 570-bp PCR product was obtained using primer pairs 105F/105R (Table S2), which amplified a 5′ segment of the *orf105* gene of pWW0. The PCR product was then cloned as a HindIII-NotI segment of the pP30D-FRT-tetO vector (26), thus generating pP30D-FRT-*tetO*-orf105. This plasmid was maintained in the E. coli CC118 strain and then passed to P. putida mt-2 strain to force recombination with pWW0. This originated strain P. putida mt-2 (pTOL-*tetO*), in which the TOL plasmid is tagged with the *tetO* array borne by the integrated construct.

### Fluorescent *in situ* hybridization.

In order to visualize *xyl* mRNAs, RNA-FISH was performed as described previously (11) with some modifications. Bacteria growing with or without aromatic effectors were fixed for 15 min at room temperature in 4% formaldehyde and 30 mM NaHPO_3_ (pH 7.5), and then keeping the samples for 30 min on ice. Cells were spun down 3 min at 4,500 × *g* and the supernatant removed. Pellets were washed with diethyl pyrocarbonate (DEPC)-treated phosphate-buffered saline (PBS) and cells resuspended in 100 μl of GTE buffer (50 mM glucose, 20 mM Tris-HCl [pH 7.5], 10 mM EDTA [pH 8]). Samples (12 μl) were next mixed with 4 μl of a solution of lysozyme and vanadyl ribonucleoside complex (VRC) such that the final concentrations of these reactions were 2.5 μg ml^−1^ and 2 mM, respectively. A 3-μl aliquot of the resulting suspension was applied onto poly-l-lysine coated coverslips and stored at room temperature until dry. Coverslips were next immersed first in methanol (10 min) and then in acetone (30 s) at –20°C. After all liquid was removed, coverslips were kept at 37°C in 10% formamide solution, 2× saline-sodium citrate buffer (SSC) in DEPC-treated water, and 2 mM VRC. After 60 min, samples were drained and 50 μl of hybridization solution (10% formamide, DEPC-treated 2× SSC, 10% dextransulphate, 2 mM VRC, 40 U RNase inhibitor, and 250 nM CAL Fluor Red 610 labeled FISH probes Stellaris, Biosearch Technologies; Table S3) was spotted onto the coverslips and incubated overnight at 42°C in a dark and humid chamber. Samples were then washed twice with 10% formamide in DEPC-treated 2× SSC for 15 min at 37°C, with DAPI (2.5 μg ml^−1^) added in the second washing step. After a brief rinse with PBS buffer, the coverslip was deposited on a slide glass treated with antifade reagent Prolong (Invitrogen) and sealed by clear nail polish. The specimen was visualized by fluorescence microscopy. To identify the intracellular position of the TOL plasmid carrying the tandem copies of the *tet* operators, DNA-FISH was performed with the 6-carboxyfluorescein (FAM)-labeled locked nucleic acid (LNA) *tetO* probe (5′ 6-FAM-CTCTATCACTGATAGGGA; Bionova). The protocol was basically the same strategy as before with RNA-FISH, but hybridization was carried out first at 95°C for 2 min to denature DNA, and then at 42°C overnight in a dark and humid chamber. Fluorescent signals exposing *xyl* mRNAs and *tetO*-tagged DNA were generated upon sequential application of RNA-FISH and DNA-FISH to the same samples.

10.1128/mBio.03685-20.3 TABLE S3 Oligonucleotide probes for RNA-FISH. Download Table S3, PDF file, 0.04 MB. Copyright © 2021 Kim et al. 2021 Kim et al. https://creativecommons.org/licenses/by/4.0/ This content is distributed under the terms of the Creative Commons Attribution 4.0 International license.

### Microscopy and image analysis.

Microscopy was performed using an Olympus BX61 instrument equipped with a 100× phase contrast objective and a DP70 camera of the same brand. Signals for red-RNA, DAPI, and green-DNA were obtained using wide field excitation with the following filters; MWIY2, U-MNU2, and U-MNIBA2, respectively. Processing of multichannel images and measurement of relative fluorescent intensities were carried out using Fiji software. Analysis of fluorescent foci representing mRNA molecules in terms of their relative cellular positions with respect to the DAPI-stained nucleoid, was carried out with the CellShape package (23). To analyze overlaps between red and blue signals, the contour lines of both channels were calculated on RGB-TIF formatted images. For this, pixel values (red/blue channels) were extracted and the resulting values normalized within the interval [0,1] in order to enable direct channel comparison regardless of any potential technical inconsistencies in measuring particular signals. This approach enabled estimation of contour lines with subpixel accuracy. The overlap values refer to the commonality between the contours for the red signals and the blue signals.
