# Supplementary material for: Subcellular Architecture of the xyl Gene Expression Flow of the TOL Catabolic Plasmid of Pseudomonas putida mt-2
Source: mBio. 2021 Feb 23;12(1):e03685-20. doi: 10.1128/mBio.03685-20 (PMC8545136; doi:10.1128/mBio.03685-20)
Supplement: FIG S1 [file mbio.03685-20-sf001.pdf]

Supplementary FIG S1. RNA-FISH experiments with *xyl* probes applied to *P. putida* mt-2 cells grown with/ without TOL aromatic effectors. Red-fluorescence labeled oligos were used that hybridized either *xylUW* or *xylX* mRNAs as indicated.

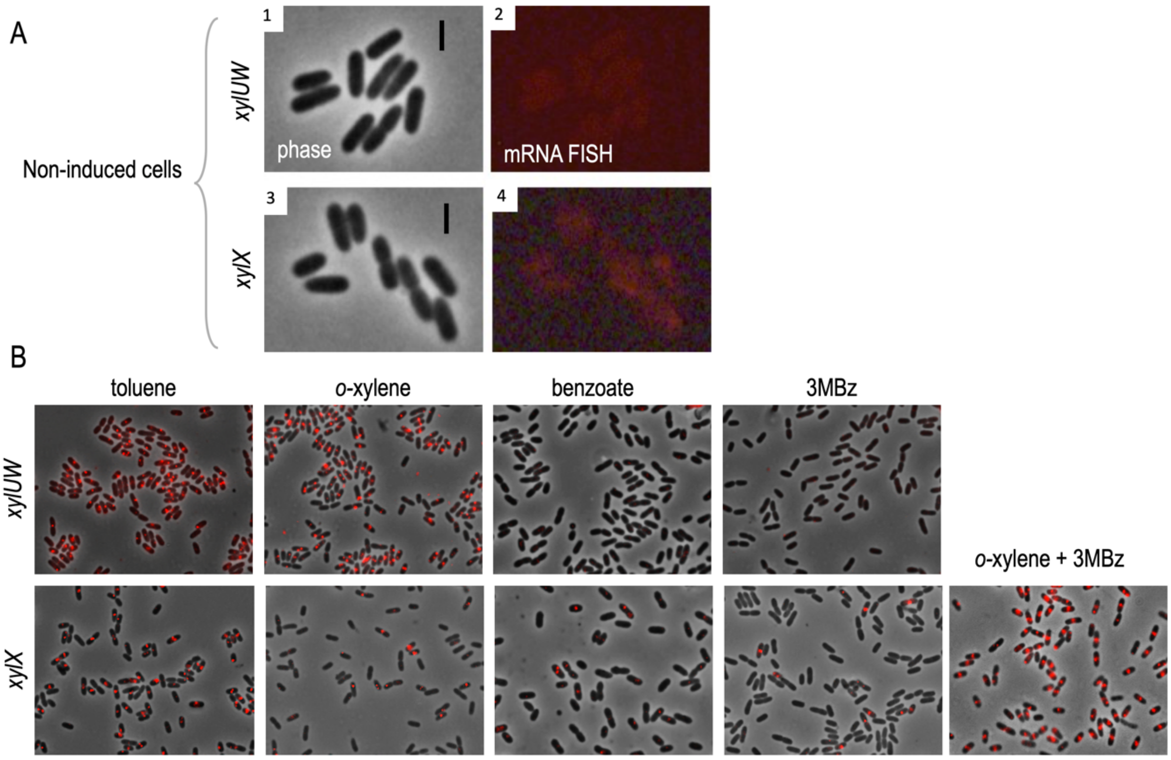

(A) Non-induced cells. Phase-contrast (panels 1 and 3); red channels (panels 2 and 4). Note that no detectable *xyl* mRNA signals were observed. Scale bar, 2.5 µm. (B) Stacked phase contrast and FISH cross-section images of *P. putida* mt-2 cells induced for 2 h with toluene, *o*-xylene, benzoate (5 mM), or 3-methylbenzoate 3MBz (5 mM). An additional culture condition with mixed effectors (*o*-xylene + 3MBz) was tested also for enhancing expression of the *lower* pathway.
