# Supplementary material for: Subcellular Architecture of the xyl Gene Expression Flow of the TOL Catabolic Plasmid of Pseudomonas putida mt-2
Source: mBio. 2021 Feb 23;12(1):e03685-20. doi: 10.1128/mBio.03685-20 (PMC8545136; doi:10.1128/mBio.03685-20)
Supplement: FIG S2 [file mbio.03685-20-sf002.pdf]

Supplementary FIG S2. DNA-FISH to visualize the pWW0 plasmid tagged with an array of *tetO* operators

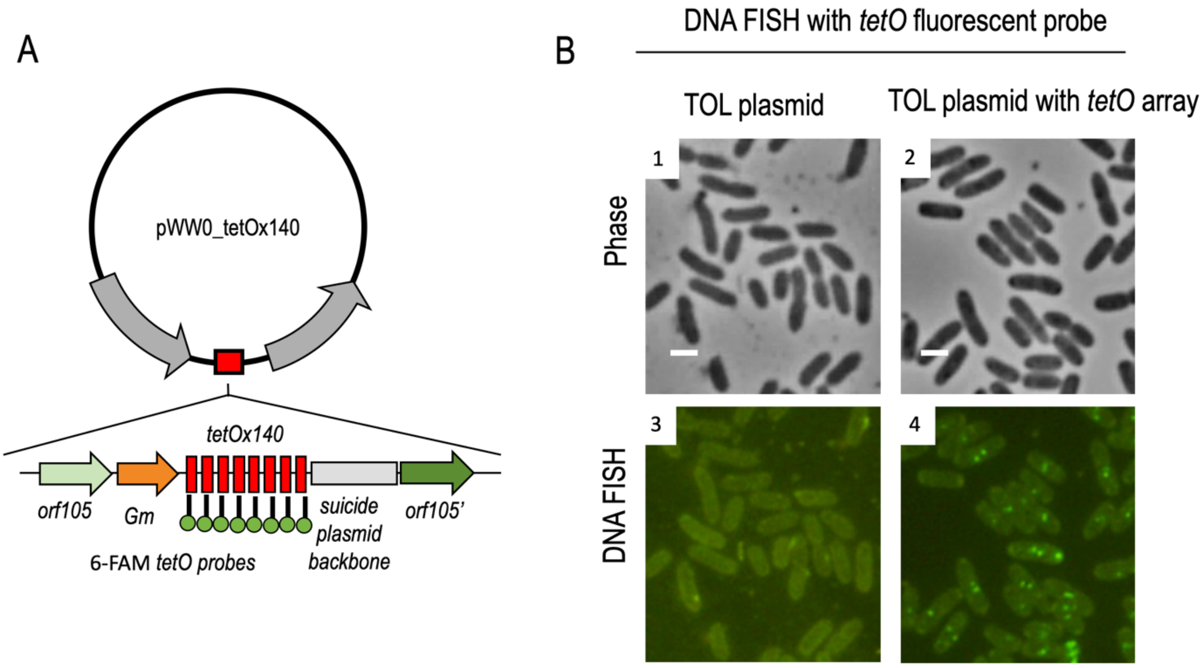

(A) Schematic representation of the modified pWW0 plasmid carrying tandemly repeated *tetO* arrays integrated in the permissive *orf105* locus of the plasmid. A 6-carboxyfluorescein (6-FAM)-labeled *tetO* probe (LNA structure) was used for the DNA-FISH procedure adopted for plasmid detection. (B) Fixed Cells carrying either the intact TOL plasmid or the variant tagged with the *tetO* array were treated with the green probe as explained in the Materials and Methods section of the main text. Phase-contrast (panels 1 and 3); DNA-green signals (panels 2 and 4). Scale bar, 1 µm.
