# Supplementary material for: Subcellular Architecture of the xyl Gene Expression Flow of the TOL Catabolic Plasmid of Pseudomonas putida mt-2
Source: mBio. 2021 Feb 23;12(1):e03685-20. doi: 10.1128/mBio.03685-20 (PMC8545136; doi:10.1128/mBio.03685-20)
Supplement: FIG S3 [file mbio.03685-20-sf003.pdf]

Supplementary FIG S3. Dual labeling of pWW0 plasmid and *xyl* mRNAs

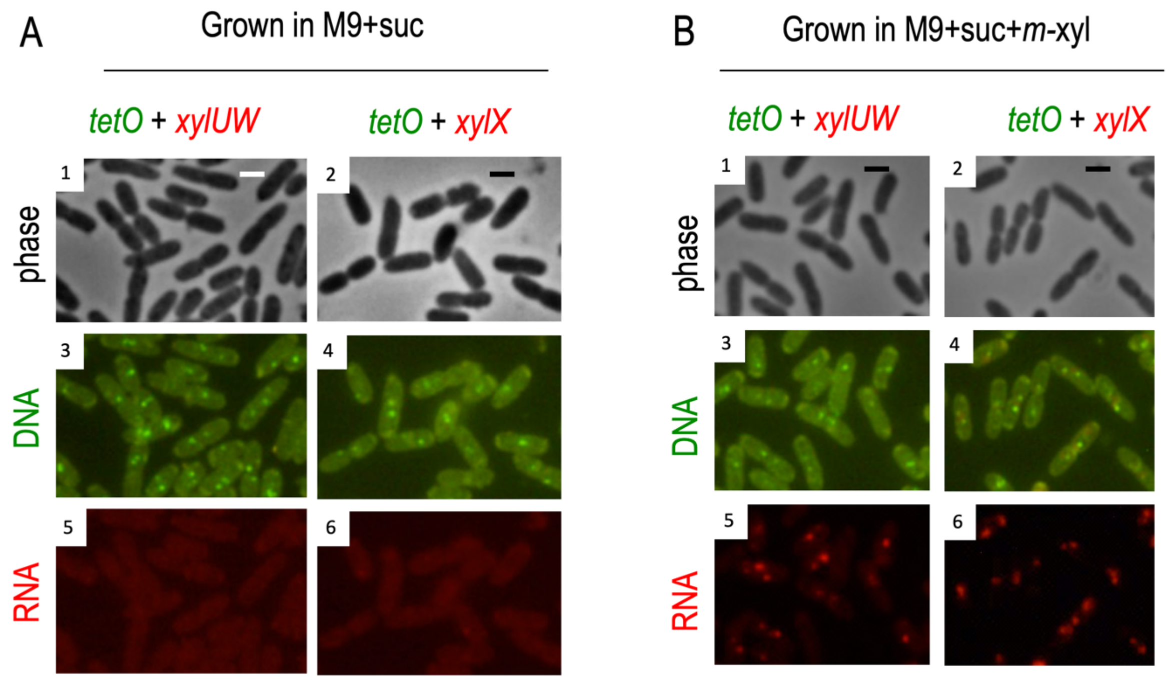

(A) Fixed *P. putida* mt-2 (pTOL-t*etO*) cells grown without effectors were sequentially subject to RNA-FISH and DNA-FISH. (B) Same cells exposed to *m*-xylene. The combined FISH approach enabled simultaneous detection of plasmid DNA (green signals; panels 3 and 4) and *xyl* mRNAs (red signals; panels 5 and 6) in the cells (panel 1 and 2) in a fashion dependent on induction of the TOL catabolic system. Scale bar, 1 μm.
