# Supplementary material for: Subcellular Architecture of the xyl Gene Expression Flow of the TOL Catabolic Plasmid of Pseudomonas putida mt-2
Source: mBio. 2021 Feb 23;12(1):e03685-20. doi: 10.1128/mBio.03685-20 (PMC8545136; doi:10.1128/mBio.03685-20)
Supplement: FIG S4 [file mbio.03685-20-sf004.pdf]

Supplementary FIG S4. Single-cell mapping of the *xyl* gene expression flow in *P. putida* mt-2 (pTOL-te*tO*) cells

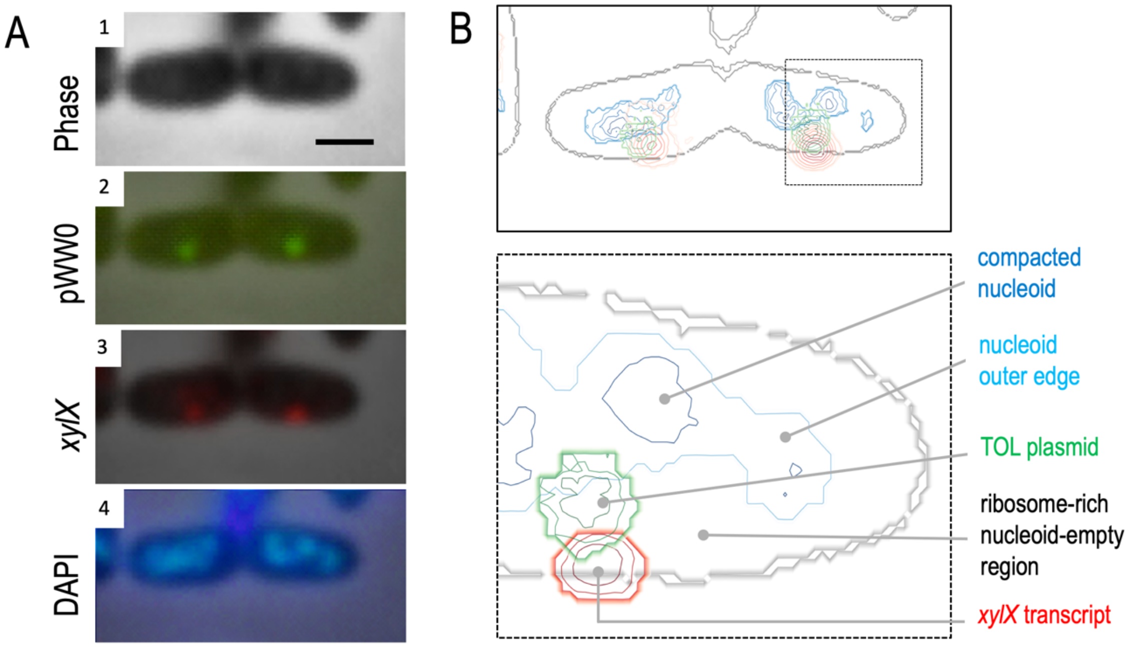

(A) A representative cell from the combined RNA-FISH and DNA-FISH procedure shows signals stemming from the pTOL-te*tO* plasmid (green; panel 2), the *xylX* mRNA (red; panel 3), and the nucleoid (blue; panel 4), respectively. Each fluorescent channel was overlaid on the phase-contrast image (panel 1). Scale bar, 1 µm. (B) All the channels, which appeared in panel A, were merged with an image analysis tool (upper panel) and the blow-up picture (lower panel), thereby exposing the subcellular localization of each molecule. Note that the plasmid DNA is attached to the nucleoid while the *xylX* transcript is found in the peripheral space of the cytoplasm.
