# Supplementary material for: Subcellular Architecture of the xyl Gene Expression Flow of the TOL Catabolic Plasmid of Pseudomonas putida mt-2
Source: mBio. 2021 Feb 23;12(1):e03685-20. doi: 10.1128/mBio.03685-20 (PMC8545136; doi:10.1128/mBio.03685-20)
Supplement: FIG S5 [file mbio.03685-20-sf005.pdf]

Supplementary FIG S5. Expression of *xylUW* mRNA with the orthogonal T7 system of and visualization of the *upper* transcripts

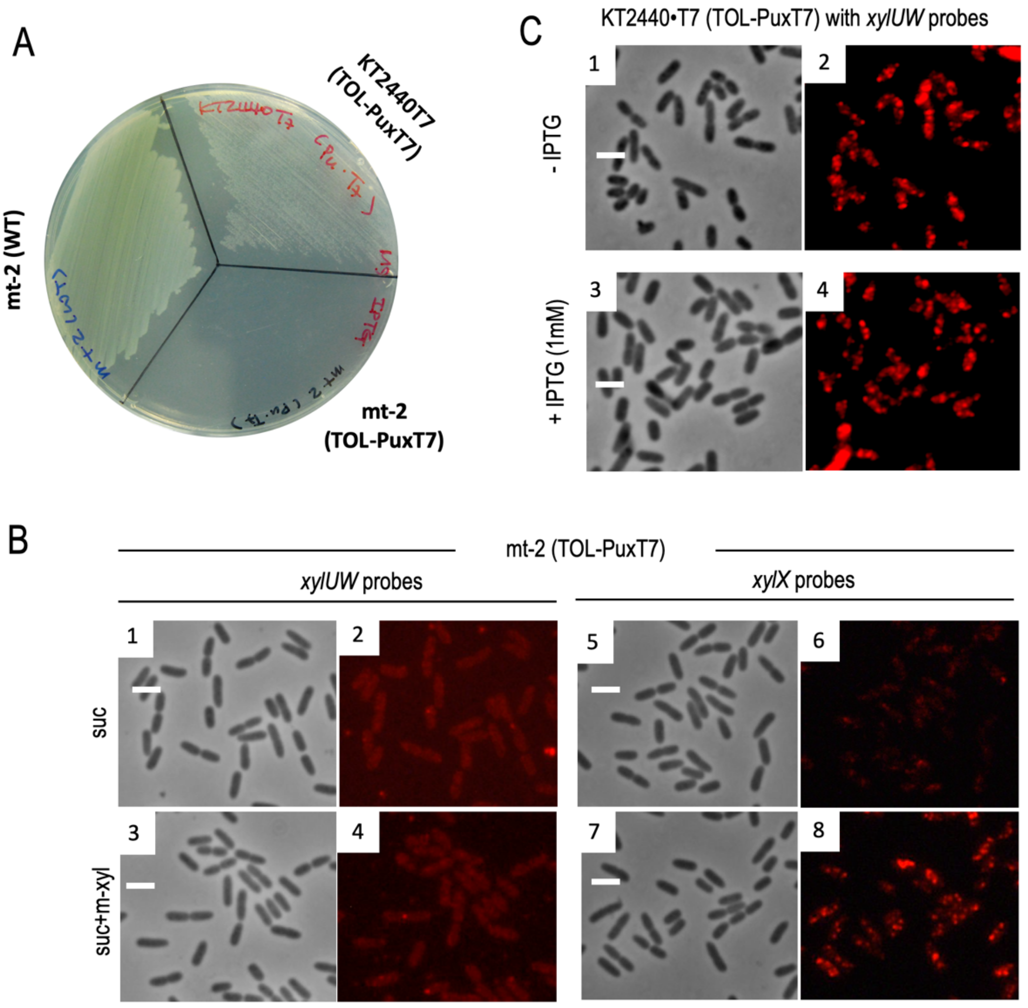

(A) *P. putida* mt-2 (wt), *P. putida* mt-2 (pTOL-PuxT7), and *P. putida* KT2440•T7 (pTOL-PuxT7) strains were streaked out on agar plates with M9 minimal medium added with 1.0 mM IPTG and exposed to saturating *m*-xylene vapors as a sole carbon source. (B) Control RNA-FISH of *P. putida* mt-2 (pTOL-PuxT7) lacking T7 RNA polymerase and grown in M9-succinate-supplemented minimal medium (panel 1, 2, 5 and 6). Same cells exposed to *m*-xylene are shown in panels 3,4,7,8. Note lack of any signal stemming from *xylUW* in contrast with spots in *m*-xylene-treated cells. This reflects transcription of the *lower* TOL operon upon overproduction of XylS caused by activation of the *Ps* promoter by XylR. (C) *xylUW* mRNA (red spots) are detected irrespective of IPTG treatment in *P. putida* KT2440•T7 (pTOL-PuxT7) strain. Scale bar, 2.5 µm.
