# Supplementary material for: Subcellular Architecture of the xyl Gene Expression Flow of the TOL Catabolic Plasmid of Pseudomonas putida mt-2
Source: mBio. 2021 Feb 23;12(1):e03685-20. doi: 10.1128/mBio.03685-20 (PMC8545136; doi:10.1128/mBio.03685-20)
Supplement: FIG S6 [file mbio.03685-20-sf006.pdf]

Supplementary FIG S6. Visualization of *xylX* mRNA in *P. putida* KT2440•T7 (pTOL-PuxT7) cells after inhibition of bacterial RNAP with rifampicin

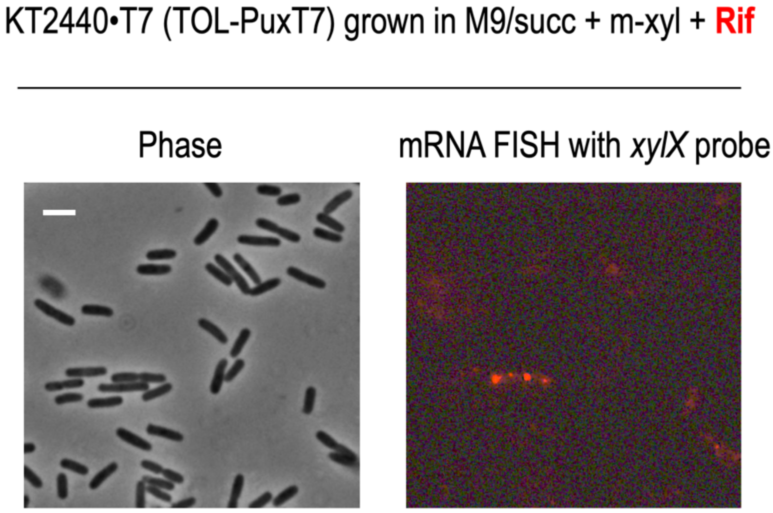

A culture of *P. putida* KT2440•T7 (pTOL-PuxT7) was treated for 2 h with *m*-xylene and rifampicin (200 μg ml^{-1}). Cells were then processed for FISH experiment with the *xylX* probe set. Expectedly, few RNA-red signals were detected due to the inhibition of bacterial RNAP by the antibiotic. Scale bar, 2.5 μm.
