# Supplementary material for: Subcellular Architecture of the xyl Gene Expression Flow of the TOL Catabolic Plasmid of Pseudomonas putida mt-2
Source: mBio. 2021 Feb 23;12(1):e03685-20. doi: 10.1128/mBio.03685-20 (PMC8545136; doi:10.1128/mBio.03685-20)
Supplement: FIG S7 [file mbio.03685-20-sf007.pdf]

Supplementary FIG S7. Visualization of *xylUW* mRNA in rifampicin-treated *P. putida* KT2440•T7 (TOL-PuxT7) cells.

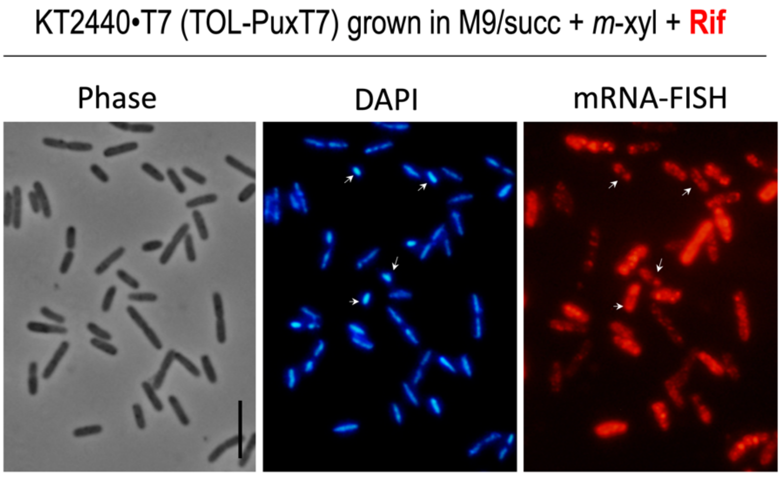

Samples (same as those shown in Supplementary Fig. S6) were probed with *xylUW*-specific oligos. Note considerable cell-to-cell variability of RNA-red spots and their diffusion through the peripheral space of the cytoplasm (marked with arrows). Scale bar, 5 μm.
