# Supplementary material for: Subcellular Architecture of the xyl Gene Expression Flow of the TOL Catabolic Plasmid of Pseudomonas putida mt-2
Source: mBio. 2021 Feb 23;12(1):e03685-20. doi: 10.1128/mBio.03685-20 (PMC8545136; doi:10.1128/mBio.03685-20)
Supplement: TABLE S1 [file mbio.03685-20-st001.pdf]

Supplementary Table S1. Description of bacterial strains and plasmids used in this study

Strain	Description	Reference
P. putida		
mt-2	*P. putida* wild type harbouring the pWW0 plasmid	(1)
PaW140	*P. putida* carrying the *upper* and the *lower* operons in the chromosome along with regulatory genes *xylR* and *xylS* genes	(2)
KT2440•T7	Gm^R, KT2440 derivative with chromosomal T7 polymerase expression system	(3)
mt-2 (pTOL-*PuxT7*)	mt-2 strain with the T7 promoter driving expression of the *upper* pathway of pWW0 plasmid	This study
KT2440•T7 (pTOL-*PuxT7*)	KT2440•T7 strain with the T7 promoter driving expression of the *upper* pathway of pWW0 plasmid	This study
mt-2 (pTOL-tetO)	mt-2 strain bearing the pWW0 plasmid tagged with an array of *tetR* operators (*tetO*)	This study
	E. coli	
HB101	Sm^R, *hsdR⁻M⁺, pro, leu, thi, recA*	(4)
DH5α*λ pir*	*λ pir* phage lysogen of DH5α	Lab collection
CC118	*F-, Δ(ara-leu)7697, araD139, Δ(lac)X74, phoAΔ20, galE, galK, thi, rpsE, rpoB*	(5)

Plasmids	Description	Reference
pRK600	Cm^R, *oriV* ColE1, *tra⁺mob⁺* of RK2, helper plasmid for mobilization in tripartite conjugations	(6)
pSW	Ap^R, *oriV* RK2, *xylS,* bearing a *Pm* → *I-sceI* transcriptional fusion	(7)
pEMG	Km^R, *ori*R6K, suicide plasmid with two I-SceI sites flanking the *lacZ*α polylinker	(8)
pEMG- PuxT7	Km^R, pEMG carrying the T7 promoter between the upstream and downstream flanking regions of the *Pu* promoter of pWW0 plasmid	This study
pP30D-FRT-tetO	Gm^R, ColE1, template for cointegration of *tetO* arrays	(9)
pP30D-FRT-*tetO*-orf105	Gm^R, pPD30D-FRT-tetO carrying a segment the *orf105* gene between *Hind*III and *Not*I sites of the plasmid	This study

REFERENCES

1. Worsey MJ, Williams PA. 1975. Metabolism of toluene and xylenes by *Pseudomonas* putida (*arvilla*) mt-2: evidence for a new function of the TOL plasmid. J Bacteriol 124:7-13.
2. Williams PA, Shaw LM, Pitt CW, Vrecl M. 1997. *xylUW,* two genes at the start of the *upper* pathway operon of TOL plasmid pWW0, appear to play no essential part in determining its catabolic phenotype. Microbiology 143 :101-107.
3. Troeschel SC, Thies S, Link O, Real CI, Knops K, Wilhelm S, Rosenau F, Jaeger KE. 2012. Novel broad host range shuttle vectors for expression in *Escherichia coli, Bacillus subtilis* and *Pseudomonas putida.* J Biotechnol 161:71-79.
4. Sambrook J, Russell D. 2001. Molecular Cloning: A Laboratory Manual. Cold Spring Harbor Laboratory Press.
5. Manoil C, Beckwith J. 1985. Tn*phoA*: a transposon probe for protein export signals. Proc Natl Acad Sci USA 82:8129-133.
6. Kessler B, de Lorenzo V, Timmis KN. 1992. A general system to integrate *lacZ* fusions into the chromosomes of gram-negative eubacteria: regulation of the *Pm* promoter of the TOL plasmid studied with all controlling elements in monocopy. Mol Gen Genet 233:293-301.
7. Wong SM, Mekalanos JJ. 2000. Genetic footprinting with mariner-based transposition in *Pseudomonas aeruginosa.* Proc Natl Acad Sci USA 97:10191-10196.
8. Martinez-Garcia E, de Lorenzo V. 2011. Engineering multiple genomic deletions in Gram-negative bacteria: analysis of the multi-resistant antibiotic profile of *Pseudomonas putida* KT2440. Environ Microbiol 13:2702-2716.
9. Vallet-Gely I, Boccard F. 2013. Chromosomal organization and segregation in *Pseudomonas aeruginosa.* PLoS Genet 9:e1003492.
