# Supplementary material for: Subcellular Architecture of the xyl Gene Expression Flow of the TOL Catabolic Plasmid of Pseudomonas putida mt-2
Source: mBio. 2021 Feb 23;12(1):e03685-20. doi: 10.1128/mBio.03685-20 (PMC8545136; doi:10.1128/mBio.03685-20)
Supplement: TABLE S2 [file mbio.03685-20-st002.pdf]

Supplementary Table S2. Oligonucleotide primers used in this study

Name	Sequences (5'-3')
PuxT7-TS1F	CGCGAATTCGTCGGATACGGCGGGCGACCG
PuxT7-TS1R	CCCTATAGTGAGTCGTATTAAGAAGACAGCCTTG ACTTTCA
PuxT7-TS2F	TTAATACGACTCACTATAGGGGACTTAAAATAAA AATAGTG
PuxT7-TS2R	CGCGGATCCCAAATGTTATAGGTAGCAAGGA
T7F	TAATACGACTCACTATAGGG
105 F	CGCAAGCTTATGAGCGATCCGGCCGTCG
105 R	CGCGGTACC GCGTCCCGCGCCGAAGCGGC
