# Supplementary material for: Subcellular Architecture of the xyl Gene Expression Flow of the TOL Catabolic Plasmid of Pseudomonas putida mt-2
Source: mBio. 2021 Feb 23;12(1):e03685-20. doi: 10.1128/mBio.03685-20 (PMC8545136; doi:10.1128/mBio.03685-20)
Supplement: TABLE S3 [file mbio.03685-20-st003.pdf]

Supplementary Table S3. Oligonucleotide probes for RNA-FISH

xylUW probe set*	*xylX* probe set*
AGCAACCAATCTGAACAGAG	AGGTGCATTGTCATGGTCAT
CCCGCTTTGAGGATATACAT	ACTATCTATATAGTCGAGCC
TCACAGACTCCAGGCGTAAC	TAGATGCCCTCGTTCTCATC
CTCAGAAAGCACTAGGCCAG	GAACATCTCGCGCTTGCAGC
CTCACCAAAATTGGTGGTCG	AATCGAACAGCCGAGGGTCG
ATAACTGCGACGAAAATGGT	TCAAAGATGTGTTTCATCTC
CCTATCACGAGAGATGAAGC	GGCGAGATAAATCCAGTTGC
CGTTGGACTGGCATCTATAA	GTTCTTCTCGGGAATCTGGC
ATTGAAGATTGATGCAGCCG	CCATCTGCGTGGTGTAATAG
CTGGGCATATAGTCGGTTGA	TGTGATGAATATCGGCTGCC
CAGGCTGGATATATCGTTGC	TCAGCTCACCATCTTTGTTG
GGTAGATGACTAAGGCTCGA	GACTGCAGGCATTGACGAAG
TAGTAATGTCGCTGCAGCTG	CACTCCTAAAGCGACAGAGC
TTCCGAGATCGACACGACTA	CCGAATTGCTGAAGGTCCAG
AAGTTCTCGGCAACAACACG	TCTTTGACCTTGAGCAGCTT
ACTTAATGCATCACATGCAG	AGTCGAAGCTGTCCGGATAG
TTATAGGTAGCAAGGACGGC	TTCTTCAGGTCGTGCGAGCC
GCGAGCATTGAATCACCTAT	GTAGGAAGCAAAGCGCGCAA
GCCCTGCTTTAGTTTTCCTT	CAGGCTGCCGAATAGAAATC
CACATCATCGACAGATAGCC	GACTCGCCGAGGAACTCTTC
AGTACTGTGGGCCTCTTTAG	GACCATGTCGATGACCTTCC
AGTCAGTACCACAGATACCG	AGCACTTCCAGACCTTCGGG
TCATTCTTTTGGCGAACTCG	AAACATAGGTACTGGAACCG
CGCTAACTTCATGACCCAAG	TGCACTTTCCAGTTGCCTTC
GATCCGACCTGTTCAATCAC	TACTGACGTGGTAGCCGTCG
CAGGCCTTAGACCTTTAACG	GCGGCGTAGTTCCAGTGAAC
AGATGACTCTCCAGACTGAC	TCTCTCAGCTTGCGCTGCTG
CGTGTAGCACGTTCCACAAG	GTCATGGCGCGAATATCATC
CGTCTTAGGACAAACATGCG	TTCAAAGGAGTAGAAACCGC
GAAGCCTCCATCAAAGTCGA	GTGCCCAGACCATCTGGTGG
TTTCCGGAACCACAACGAAA	GGCGGTTTTTCGGGTCACCC
CGCAACCAGAAACAAGAACA	CGATCTCGCTCGGCGAACAG
TGCAATGTTCATAAGTCCGA	TTCACCAAACTCGCTGGCTA
GATGAATGTCCGGTGCAATG	GAGACGCCGATCATCCAGTC
ATTTTGGCTGCTGCAGTGAG	GAACTGGTCCATCAGGTAGA
GCATTAATGCATTCATCCGC	GACGGGTGATACGCAACTGC

CGCACAGTCTTATAAACGCT	GATTTCGGTTCTATCCACCG
GCTTGCCCAGAATAGTCAAT	CTCGGCGTTTCGCCTTTGGG
AATAAGCTCCTTTAACGCCG	GTCCTCGTACTGACGGACAC
AAATATCATGTGACGGGACG	CCATGCCGCTGACATTGAAG
CCACGTACAATAAGGCCTTT	GGAATTCCTCCAGGTCGTCC
ATCTTTCATACAGACGCCTG	CACGGGACATGTCGTTCATC
CCAATAAGCTAGTTGAACGC	CCCTCGATCCAGTGTTTGGC

*Each oligo was labeled with the fluorophore CAL Fluor Red 610
